# Efficacy of intrathecal baclofen bolus on neuropathic pain in patients with spinal cord injury: A protocol for systematic review and meta-analysis

**DOI:** 10.1097/MD.0000000000020484

**Published:** 2020-06-19

**Authors:** Shou-feng Wang, Zeng-mian Wang, Wei-dong Song, Zhao-chen Tang, Ying Chai

**Affiliations:** a First Ward of Orthopedics Department; b Third Ward of Neurology Department, First Affiliated Hospital of Jiamusi University, Jiamusi; c Department of Orthopedics, Second Affiliated Hospital of Mudanjiang Medical University, Mudanjiang; d School of Clinical Medicine, Jiamusi University, Jiamusi, China.

**Keywords:** efficacy, intrathecal baclofen bolus, neuropathic pain, spinal cord injury

## Abstract

**Background:**

This study will explore the efficacy and safety of intrathecal baclofen bolus (IBB) on neuropathic pain (NPP) in patients with spinal cord injury (SCI).

**Methods:**

All potential literatures of IBB on NPP in patients with SCI will be searched from the following electronic databases from inauguration to the January 31, 2020: PUBMED, EMBASE, Cochrane Library, Web of Science, Chinese Scientific Journal Database Information, WANGFANG, and China National Knowledge Infrastructure. In addition, we will search other sources, such as dissertations and reference lists of included trials. There are no restrictions of language and publication status in searching all literature sources. The quality of each eligible trial will be assessed using Cochrane risk of bias tool, and publication bias will be checked using a funnel plot and Egger test. Statistical analysis will be conducted using RevMan 5.3 software.

**Results:**

This study will scrutinize the efficacy and safety of IBB on NPP in patients with SCI through pain intensity of NPP, spasticity, walking ability, health-related quality of life, duration of stay at hospital (days), incidence of adverse event, and mortality rate.

**Conclusions:**

The findings of this study will present helpful evidence to judge whether IBB is effective on NPP in patients with SCI or not.

**Study registration number:**

INPLASY202040192.

## 1 Introduction

Spinal cord injury (SCI) is a common neurological disorder in adult population, with the ratio of male-to-female is around 2:1.^[1–4]^ It is estimated that its incidence is about 40 to 80 new cases per million people annually from all causes.^[5–7]^ Patients with SCI often experience paralyzed muscles, atrophy, walking disability, spasticity, and neuropathic pain (NPP).^[8–10]^

A variety of studies have explored the efficacy and safety of intrathecal baclofen bolus (IBB) on NPP in patients with SCI.^[11–21]^ However, it is plausible to hypothesize that IBB can reduce NPP in patients with SCI. In addition, its reports on NPP relief are rare at literature levels. Thus, the purpose of this study is to compare the efficacy and safety of IBB on NPP after SCI with those of other treatments.

## 2 Methods

### 2.1 Study registration

This protocol was registered on INPLASY202040192. We report this study based on the guidelines of the Preferred Reporting Items for Systematic Reviews and Meta-Analysis Protocol statement.^[22,23]^

### 2.2 Ethics and dissemination

It is not necessary to provide ethical approval, because this study only extracts data from included study. It will be submitted and published in a scientific peer-reviewed journal or a conference meeting.

### 2.3 Criteria for including studies

#### 2.3.1 Types of studies

This study will only include randomized controlled trials (RCTs) of IBB on NPP in patients with SCI. Any other studies including quasi-RCTs will be excluded from this study.

#### 2.3.2 Types of interventions

All patients in the experimental group underwent IBB alone as their management for NPP.

All participants in the control group received any treatments, such as alternative medicine, massage, or any other interventions. However, we will exclude patients who also taken IBB.

#### 2.3.3 Types of patients

Any SCI patients who were diagnosed as NPP will be included in this study. No restrictions upon race, gender, age, severity, and duration of SCI and NPP will be applied to this study.

#### 2.3.4 Types of outcome measurements

Primary outcome is pain intensity of NPP, as measured by Neuropathic Pain Symptom Inventory or any other relevant pain scales.

Secondary outcomes are spasticity (as assessed by Modified Ashworth Scale or other associated scales), walking ability (as checked by 10 m-Walk Test or other tools), health-related quality of life (as identified by 36-Item Short Form Survey or other questionnaires), duration of stay at hospital (days), incidence of adverse event, and mortality rate.

### 2.4 Data sources and search

A comprehensive search will be conducted in the following electronic databases from their onset to the January 31, 2020: PUBMED, EMBASE, Cochrane Library, Web of Science, Chinese Scientific Journal Database Information, WANGFANG, and China National Knowledge Infrastructure. We will not utilize any limitations of language and publication date to the literature search. The sample of search strategy for PUBMED is presented in Table 1. We will adapt similar search strategies with specifics to other electronic databases.

**Table 1:**
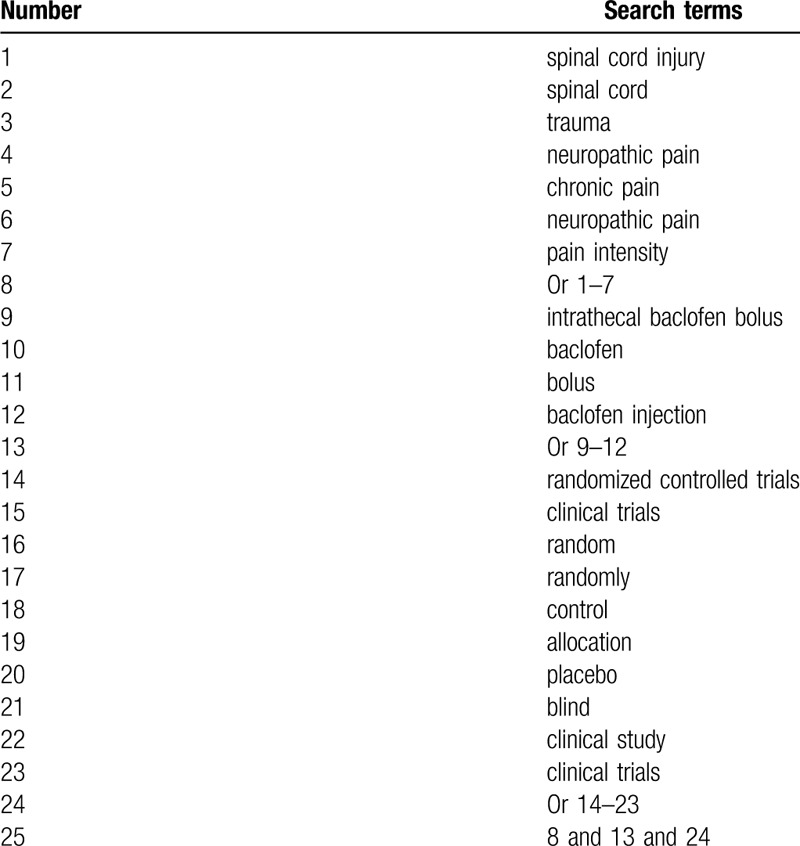
Search strategy of PUBMED.

In addition to the above electronic databases, we will also check other resources, including dissertations and reference lists of qualified studies.

### 2.5 Data collection and analysis

#### 2.5.1 Study selection

**Figure 1:**
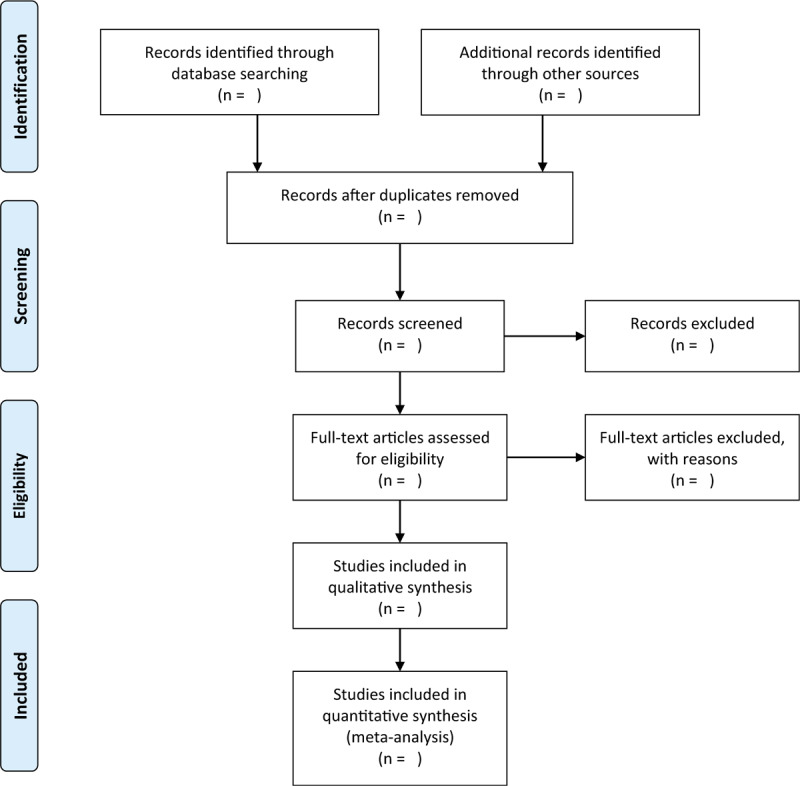
Flow chart of study selection process.

All study records will be managed using Endnote X7 and all duplications will be removed. Titles and abstracts of all studies will be identified by 2 independent authors to investigate eligible studies in accordance with the eligibility criteria, and all unrelated records will be removed. Then, we will obtain all remaining studies with full-texts and will cautiously examine all inclusion criteria to determine whether they fulfill and should be included in this study. Any disagreements will be settled down with the help of a third author via discussion. We will present the study selection process in the flow chart (Fig. 1). The excluded reasons for all removed studies will be recoded.

#### 2.5.2 Data collection

Data will be collected from the included trials by 2 independent authors using an advance-designed data extraction sheet. Any inconsistencies will be resolved by discussion with another experienced author. We will collect data of title, first author, publication date, country, demographic characteristics of patients (such as age, sex, race, et al), trial setting, sample size, trial methods (such as randomization, blind, et al), interventions, comparators, outcome variables, results, findings, follow-up data, and conflict of interest.

#### 2.5.3 Missing data dealing with

If there is unclear or missing data, we will contact original authors to request it. If such data is not obtained, we will only analyze available data using an intention-to-treat analysis. In addition, we will discuss its potential impact on the study findings.

#### 2.5.4 Risk of bias assessment

Two authors will independently appraise the study quality of each eligible trial using the internationally recognized Cochrane risk of bias tool for assessing RCTs. It consists of 7 aspects, and each item is classified as low, unclear, or high risk of bias. Any discrepancies will be solved by a third author, and consensus is reached.

#### 2.5.5 Subgroup analysis

We will carry out subgroup analysis to find out possible reasons of the substantial heterogeneity according to the different types of treatments, controls, and outcome measurements.

#### 2.5.6 Sensitivity analysis

We will preside over sensitivity analysis to identify the robustness and stability of study findings by excluding low quality trials.

#### 2.5.7 Reporting bias

We will test reporting bias using funnel plot and Egger regression test when more than 10 eligible trials are included in this study.^[24,25]^

#### 2.5.8 Quality of evidence

Grading of Recommendations Assessment, Development, and Evaluation^[26]^ will be used for assessing the quality of evidence for the primary outcome by 2 independent authors. Any inconsistencies will be solved by a third author via discussion.

### 2.6 Data synthesis

This study will use RevMan 5.3 software to conduct all statistical analysis. Continuous outcome values will be expressed as mean difference or standardized mean difference and 95% confidence intervals (CIs), and dichotomous outcome values will be explicated as risk ratio and 95% CIs. Statistical heterogeneity among qualified trials will be performed by *I*^*2*^ statistics. *I*^*2*^ ≤ 50% suggests low heterogeneity, and a fixed-effects model will be used for synthesizing outcome data. *I*^*2*^ > 50% states considerable heterogeneity, and a random-effects model will be employed for pooling outcome data. If sufficient data is collected and low heterogeneity is identified, we will carry out meta-analysis based on the similar study characteristics, types of interventions and controls, and outcome measurements. If significant heterogeneity is checked, we will perform subgroup analysis to explore its possible reasons of the considerable heterogeneity.

## 3 Discussion

It is reported that IBB can be a promising therapy to relieve NPP in patients with SCI.^[11–22]^ However, no systematic study reported and summarized the available evidence in this population. The present study will examine and synthesize the evidence for the efficacy and safety of IBB on NPP after SCI with those of other treatments. The results of this study may help clinicians choose the best options for the treatment of NPP in patients with SCI, as well as provide evidence for decision-making of guidelines.

## Author contributions

**Conceptualization:** Zhao-chen Tang, Ying Chai.

**Data curation:** Shou-feng Wang, Zeng-mian Wang, Wei-dong Song, Ying Chai.

**Formal analysis:** Zeng-mian Wang, Wei-dong Song.

**Funding acquisition:** Ying Chai.

**Investigation:** Ying Chai.

**Methodology:** Zeng-mian Wang, Wei-dong Song, Zhao-chen Tang.

**Project administration:** Ying Chai.

**Resources:** Shou-feng Wang, Zeng-mian Wang, Wei-dong Song, Zhao-chen Tang.

**Software:** Shou-feng Wang, Zeng-mian Wang, Wei-dong Song, Zhao-chen Tang.

**Supervision:** Ying Chai.

**Validation:** Shou-feng Wang, Zeng-mian Wang, Ying Chai.

**Visualization:** Shou-feng Wang, Wei-dong Song, Zhao-chen Tang, Ying Chai.

**Writing – original draft:** Shou-feng Wang, Zeng-mian Wang, Wei-dong Song, Zhao-chen Tang, Ying Chai.

**Writing – review & editing:** Shou-feng Wang, Zeng-mian Wang, Zhao-chen Tang, Ying Chai.

## References

[1] Shiao R Lee-Kubli CA Neuropathic pain after spinal cord injury: challenges and research perspectives. Neurotherapeutics 2018;15:635–53. 29736857 10.1007/s13311-018-0633-4 PMC6095789

[2] Rabinstein AA Traumatic spinal cord injury. Continuum (Minneap Minn) 2018;24:551–66. 29613899 10.1212/CON.0000000000000581

[3] Ahuja CS Wilson JR Nori S Traumatic spinal cord injury. Nat Rev Dis Primers 2017;3:17018. 28447605 10.1038/nrdp.2017.18

[4] Rogers WK Todd M Acute spinal cord injury. Best Pract Res Clin Anaesthesiol 2016;30:27–39. 27036601 10.1016/j.bpa.2015.11.003

[5] Burke D Fullen BM Stokes D Neuropathic pain prevalence following spinal cord injury: a systematic review and meta-analysis. Eur J Pain 2017;21:29–44. 27341614 10.1002/ejp.905

[6] Singh A Tetreault L Kalsi-Ryan S Global prevalence and incidence of traumatic spinal cord injury. Clin Epidemiol 2014;6:309–31. 25278785 10.2147/CLEP.S68889 PMC4179833

[7] Furlan JC Sakakibara BM Miller WC Global incidence and prevalence of traumatic spinal cord injury. Can J Neurol Sci 2013;40:456–64. 23786727 10.1017/s0317167100014530

[8] Karatas G Metli N Yalcin E The effects of the level of spinal cord injury on life satisfaction and disability. Ideggyogy Sz 2020;73:27–34. 32057201 10.18071/isz.73.0027

[9] Argetsinger LC Singh G Bickel SG Spinal cord injury in infancy: activity-based therapy impact on health, function, and quality of life in chronic injury. Spinal Cord Ser Cases 2020;6:13. 10.1038/s41394-020-0261-1 PMC7064539 32157078

[10] Massa LM Hoffman JM Cardenas DD Validity, accuracy, and predictive value of urinary tract infection signs and symptoms in individuals with spinal cord injury on intermittent catheterization. J Spinal Cord Med 2009;32:568–73. 20025153 10.1080/10790268.2009.11754562 PMC2792463

[11] Kumru H Benito-Penalva J Kofler M Analgesic effect of intrathecal baclofen bolus on neuropathic pain in spinal cord injury patients. Brain Res Bull 2018;140:205–11. 29782907 10.1016/j.brainresbull.2018.05.013

[12] Vaidyanathan S Oo T Soni BM Severe, protracted spasm of urinary bladder and autonomic dysreflexia caused by changing the suprapubic catheter in a cervical spinal cord injury patient: treatment by a bolus dose and increased total daily dose of intrathecal baclofen. Clin Med Insights Case Rep 2016;9:119–21. 28008298 10.4137/CCRep.S39117 PMC5156549

[13] Kumru H Kofler M Effect of spinal cord injury and of intrathecal baclofen on brainstem reflexes. Clin Neurophysiol 2012;123:45–53. 22030139 10.1016/j.clinph.2011.06.036

[14] D’Aleo G Rifici C Kofler M Favorable response to intrathecal, but not oral, baclofen of priapism in a patient with spinal cord injury. Spine (Phila Pa 1976) 2009;34:E127–9. 19179913 10.1097/BRS.0b013e31818d04ff

[15] Elovic E Kirshblum SC Managing spasticity in spinal cord injury: safe administration of bridge boluses during intrathecal baclofen pump refills. J Spinal Cord Med 2003;26:2–4. 12830961 10.1080/10790268.2003.11753652

[16] Yu JG Observation of the therapeutic effect of baclofen on myospasm after spinal cord injury. Chin Foreign Med Sci 2013;32:120–1.

[17] Li D Liu W Wang JC Observation of the therapeutic effect of baclofen on myal spasm after spinal cord injury. Modern J Integr Trad Chin West Med 2011;20:39–40.

[18] Lin SD Qian SQ Zhang YC Evaluation and clinical progress of spasticity after spinal cord injury. Chin J Orthop Surg 2008;6:435–7.

[19] Liu GL Li JJ Zhou HJ Observation of the therapeutic effect of Baclofen on spasticity after spinal cord injury. Chin Rehab Theory Pract 2008;1:77–8.

[20] Du DP Xu YM Jiang W Effect of intrathecal injection of baclofen on the content of excitatory amino acids in spinal cord of rats with acute incisional pain. Shanghai Med 2007;7:500–3.

[21] Hu AL Liang XY Zhang BY Baclofen relieves muscle spasm after spinal cord injury. Chin J Clin Rehab 2003;22:3145.

[22] Shamseer L Moher D Clarke M PRISMA-P Group. Preferred reporting items for systematic review and meta-analysis protocols (PRISMA-P) 2015: elaboration and explanation. BMJ 2015;349:g7647. 10.1136/bmj.g7647 25555855

[23] Moher D Shamseer L Clarke M Preferred reporting items for systematic review and meta-analysis protocols (PRISMA-P) 2015 statement. Syst Rev 2015;4:1. 25554246 10.1186/2046-4053-4-1 PMC4320440

[24] Sutton AJ Duval SJ Tweedie RL Empirical assessment of effect of publication bias on meta-analyses. BMJ 2000;320:1574–7. 10845965 10.1136/bmj.320.7249.1574 PMC27401

[25] Egger M Davey Smith G Schneider M Bias in meta-analysis detected by a simple, graphical test. BMJ 1997;315:629–34. 9310563 10.1136/bmj.315.7109.629 PMC2127453

[26] Guyatt GH Oxman AD Vist GE GRADE: an emerging consensus on rating quality of evidence and strength of recommendations. BMJ 2008;336:924–6. 18436948 10.1136/bmj.39489.470347.AD PMC2335261

